# Supplementary material for: Caveat emptor, computational social science: Large-scale missing data in a widely-published Reddit corpus
Source: PLoS One. 2018 Jul 6;13(7):e0200162. doi: 10.1371/journal.pone.0200162 (PMC6034852; doi:10.1371/journal.pone.0200162)
Supplement: S1 File — Authors of all cited works were solicited for opportunities to respond to our findings—Jack Hessel, Lillian Lee, David Mimno, and Chenhao Tan jointly have provided a response. (PDF) [file pone.0200162.s001.pdf]

Hi Devin and Nate,

We would first like to thank you again for bringing this issue to our attention. Your systematic evaluation was quite thorough and much appreciated. As social media datasets continue to increase in size, more work like yours should be completed, and we very much appreciate your constructive and careful efforts. We definitely agree that "all datasets have biases, no matter how complete we wish them to be;" more explicit qualifications of this dataset's shortcomings in future work would be appropriate. While we do think that this dataset's ambition, scale, cleanliness, and reach outweigh the downsides, i.e., we don't think that scraping errors should preclude future work, users of this dataset should be explicit about the possible caveats in working with it.

1. We could not agree more that the responsibility of data validation and cleaning should not fall to Jason Baumgartner. He has done a tremendous amount of work and we are all in his debt. Future data collection and cleaning should not automatically fall to him.
2. Although we began with RedditAnalytics, as we acknowledged in Tan & Lee 2015, we constructed our (Tan & Lee 2015, from which Hessel, Tan and Lee 2016, Hessel et al. 2016, and Hessel, Lee, and Mimno 2017 were derived) reddit submissions dataset prior to Jason B. releasing the full post set on pushshift.io, and our dataset differs from it. In fact, prior to our experimental work we ran a number of consistency and validation tests on the initially extracted data, contacted Jason to alert him of some inconsistencies that were revealed by our tests, and re-scraped some systematically missing submission data (for example, the README[1] for our data release describes the re-scraping of all reddit posts for a month).
3. That being said, while the systematic biases you discover may differ from the pushshift.io post set to the one we use, it's possible that the set we are using also has similar biases. We will go back and check properties of our set. When we were constructing these datasets, we were aware of a small amount of potential missing data, though we judged that the amount of it was relatively insignificant in light of several other dataset characteristics. There are several well-known shortcomings of reddit data, including the issue of deleted posts. For example, in the dataset we use, there are 101M submissions, of which around 25M are deleted with no author information available. The missing comments/submission values you report (.043% and .65% respectively) were similar to the values we discovered in our own validations. Given that deleted posts (~25%) are a well-known shortcoming of any study using reddit data, we estimated that the small amount of missing data due to scraping errors (~1%) was less significant, and was a factor we ignored after correcting for large, systematic problems. In some cases, we ran additional experiments controlling for the likely more impactful factor of deleted posts, e.g., footnote 18 in Hessel, Lee, and Mimno 2017.[2] In Tan and Lee 2015, since we knew of such issues, we should not have glossed over them by being so free in

[1] https://chenhaot.com/data/multi-community/README.txt
[2] https://arxiv.org/pdf/1703.01725.pdf

saying “all posts”; similar problematic shorthand in follow-up work should not have been used.

4. Even after Jason's latest re-scraping, though, there still are some inexplicable findings that call into question the possibility of absolute completeness of a corrected version of this dataset. Here are few observations we have made based on a reading of your work and some previous analyses we had conducted.
    a. In the new reddit 0-10M post scrape, only 955K (9.5%) of posts are found. Here are some (base36, base10) submissions that were found within the first 10M ids: (2pjh7, 4550875), (2pjhg, 4550884), (2pjhq, 4550894); under the one-id-per-post metric, there clearly are still a significant number of missing posts -- perhaps up to 90%. If one attempts to access posts in between these values (e.g., (2pjh8, 455086)) either via the browser[3] or via the API, a 404 error is returned (as of 3/9/2018). We've run into similar questions before, and attempted to find out why posts might be missing.
    b. To be concrete: after reading your work, we conducted a test with our own set. How many of the "gaps" in our data could be filled by re-querying the API? Using a random test set of 400 uniformly sampled post ids in the base36-sequential gaps in our set, we found that only 53/400 (13%) were accessible -- the remaining 347 (87%) posts were errors (we checked to see if this was reproducible in the browser -- for the posts we checked, it was). It's not entirely clear as to what this means. This observation invites many questions, e.g., are posts always assigned a continuous sequence of base36 ids? Can reddit moderators fully delete posts? Does the availability of posts according to the API change over time? Were the 13% of found posts unavailable for some reason in 2014? Have practices remained consistent since 2007? Do these potentially missing posts mean that future reddit studies are not to be trusted?
    c. Among the 347 failed API queries, there was a mixture of 404 and 403 errors (specifically: 289 404 errors (generally -- these were from earlier posts), 58 403 errors (generally -- these were from later posts)). According to the reddit python API documentation, a 403 error can be caused by a variety of circumstances, e.g., if the subreddit is private and the requesting user doesn't have access to it. According to the reddit python API, a 404 error occurs if the resource does not exist; it's plausible that a non-existent resource could be caused by an id that was never allocated, though it's hard to say why this happens. We have attempted to search through cached internet resources in search of missing posts (e.g., the internet archive) to no avail.
    d. We have also noticed some odd patterns in early reddit ids that could plausibly be indicative of future discontinuities, particularly if the id assignment scheme has remained consistent over time. For example, when sorting the very first posts made to reddit according to Jason's latest scrape by the created_utc field, the post ids up to post 28128 appear to be in base 10 (in base36, they are highly

[3] http://reddit.com/r/reddit.com/comments/1l3tm/broken_link/

inconsistent). Then, the next post's id is "sqh" which would be post 37241, made 71 minutes after. Scrolling through this list reveals many more discontinuities; in time, the following post sequence appears: 101x, 5yb9e (200 seconds later), 5yb98 (300 seconds later), 102c (50 seconds later). These oddities continue, too; for instance, here is a post[4] from the new re-scraping that is impossible -- the comment was made 4 years prior to the post.

e. Anecdotally, it seems that most of the oddities in the dataset occur before 2008. Jan. 2008 is when Reddit started to allow users to create their own communities. We have found that most of the missing data after 2008 are due to 403 errors, which means that these submissions are likely not publicly available. As a result, we think that the Reddit dataset curated by Jason B. is amenable to studies focused on user-created communities, particularly in comparison to other publicly available datasets.

In closing, we think that your work has raised an interesting research question: given that it's generally impossible to know if one has collected a "complete" set of information (be it posts by a reddit user, a user's tweets, wikipedia talk comments, etc.) are there methods of evaluation that simultaneously allow us to ask interesting questions while making sure observations are insensitive to often-overlooked (and, in the case of reddit, potentially un-avoidable) scraping gaps? We would be happy to discuss this topic going forward!

And, one bureaucratic point: if you found this email (or followup conversations) helpful, we would be happy about that, but ask that if you were to acknowledge us in any paper or the like, please include a disclaimer that we do not necessarily agree with every point made in your paper. (Obviously, you don't have to acknowledge us at all if this wasn't helpful!)

Signed (alphabetically),

Jack Hessel
Lillian Lee
David Mimno
Chenhao Tan

[4] http://reddit.com/r/reddit.com/comments/1l3tm/broken_link/
